# Medicine

**Published:** 1902-09

**Authors:** R. Shingleton Smith


### Progress of the Medical Sciences.

### MEDICINE.

The subject of **Thrombosis** has of late derived a new interest from the recognition of its association with various infective processes, and to the new light as to its pathology which the demonstration of various micro-organisms has given. "The word 'thrombosis' conjures up before our minds not those benign effects, but some of those disastrous results which we so often see following upon the untimely clotting of blood within the vessels. We think of pains and long-drawn-out disabilities, of extended periods of rest and idleness, perhaps of permanent lameness or paralysis, or even of sudden death, or the *ante-mortem* agony, all of them so often resulting from thrombosis. Indeed, it plays almost as prominent a part in disease and death as in the preservation of health and life; perhaps we hardly realise how large a part it plays . . . how enormous an advance in practical medicine it will be when thrombosis will be known only as a beneficent phenomenon and when we are able to prevent its occurrence as a baneful process."[1] Phlegmasia alba dolens, milk leg, phlebitis, commonly considered to be a somewhat rare occurrence, was described by Paget[2] as not unusual after typhoid;

[1] Pearce Gould, "Thrombosis," *Lancet*, 1902, i. 1583.

[2] *Selected Essays*, 1902, p. 212.

and Sir James explained it as an inflammatory thickening and swelling of the walls of the vein with consequent narrowing of the canal, occurring commonly when the temperature has become normal and constant, but not usual during the continuity of the fever. Murchison described it as occurring in about 1 per cent. of the cases of typhoid. Da Costa gives a very different record, for he found in a series of 135 cases[1] that it occurred in 14 per cent. of cases, which were mostly of a severe type, during the period of convalescence, one case on the fortieth day, another on the fiftieth, another on the fifty-ninth day. Of the 18 cases, 3 were in the left leg, 2 in the right leg alone, and 13 in both legs. In two cases the thrombosis occurred in a relapse whilst the temperature was still high. The long-debated question as to the occurrence of a primary thrombosis in a healthy vein, or the presence of phlebitis, has been solved by the demonstration of bacilli in the clot, the clotting being due to the bacilli in the sluggish circulation of the infected blood.[2] It is now generally admitted that thrombosis is commonly due to an infective arteritis or phlebitis, that the infection may be due to a great variety of micro-organisms, and that the occurrence of thrombosis is evidence of an infective condition in the great majority of cases.

Cases of peripheral venous thrombosis in pneumonia have been reported by Walter H. Steiner, M.D.[3] He alludes to the fact that it is common in certain infective diseases, such as typhoid and influenza, also following some abdominal operations, but that it is rare in pneumonia.

Da Costa's case, reported in 1894, was then considered to be unique; but in 1898 he reported two others, making three cases in a series of 500, and the following conclusions were drawn:—1. Rarity of thrombosis in pneumonia; only 38 cases could be collected. 2. In 27 out of the 32 it occurred during convalescence, and must be regarded as a sequel rather than a complication of the pneumonia; and 3. The lower extremities were always the parts involved, the left leg in 16, the right in 10, and both in 7 cases.

In malaria, syphilis, tuberculosis, puerperal, and other septic states, thrombosis is by no means rare, as also is the case in chlorosis, when it is likely to be due to mechanical and other than bacterial conditions.

Leichtenstern found it 11 times in 1653 chlorotics, and that it commonly affected both lower limbs. In a collected series of 86 cases the lower limbs were affected 48 times, and the cerebral vessels 29 times.

In accordance with modern views as to the etiology of rheumatic conditions, an infective thrombosis is likely to be of occurrence in rheumatic states. Sansom[4], however, states

[1] *Boston M. & S. J.*, 1899, cxl. 273. [2] *Hanshalter. Merc. Med.*, Sept., 1893.
[3] *Johns Hopkins Hosp. Bull.*, 1902, xiii. 130.
[4] *Twentieth Century Practice of Medicine*, 1896, vol. iv., p. 639.

that it is not known to exist at all; but if the conclusions of Poynton and Paine[1] are correct, an infective thrombosis of rheumatic origin should now and then be recorded.

Two such cases have recently been observed by myself. A man, æt. 62, ill three months with rheumatic pyrexia and two relapses, with many joints swollen, and relieved by salicylates, but with no obvious heart defect, became hemiplegic on right side and aphasic: after one week he had recovered some power in the arm and could speak a few words; but a week later became more and more somnolent and died without a fresh attack of any kind, and with no return of pyrexia or any heart symptoms.

The other case is that of a man, aged 40, whose illness commenced in February with left sciatica; a month later he had pyrexia, with swelling of the right thigh and calf, due to thrombosis of the femoral vein; afterwards similar swelling on the left side; one month later intercostal pains with pleural effusion at the left base occurred, and ten days later pleural effusion at the right base. Meanwhile the rheumatic symptoms, with more or less persistent pyrexia, continued for many weeks, and the convalescence was unusually protracted, as is usually the case with thrombosis of both femoral veins.

Such cases as these naturally suggest the idea that the thrombosis is of infective origin, and resembles that following typhoid, pneumonia, and puerperal conditions, and it gives an argument in favour of the views advocated by Poynton and Alexander Paine[2], showing that all the varieties of rheumatism, even the osteo-arthritic type of rheumatoid arthritis, and the malignant endocarditis of rheumatic origin, are of an infective character.

A paper by Dr. W. H. Welch[3], of Baltimore, directs attention to venous thrombosis as a complication of cardiac disease. He had been able to collect a series of 27 cases, and in the majority the location of the thrombus was in the veins of the neck and upper extremities. In one case, reported by the author, the streptococcus pyogenes was found, and it was thought that the thrombosis was, even in such cases as these, usually of micro-organismal origin. In another such case, reported by Dr. D. B. Lees[4], thrombosis of the brachial veins had been present.

It seems likely that further evidence will tend to show that the microbial origin of thrombosis is much more common than has been supposed, and is of almost equal importance with, perhaps more so than, the various mechanical conditions connected with the heart and blood-vessels, which are well-recognised causes of clotting in the veins and elsewhere.

[1] *Bristol. M.-Chir. J.*, 1901, xix. 136.
[2] *Brit. M. J.*, 1902, i. 79, 895.
[3] *Brit. M. J.*, 1900, i., Epitome, p. 85. [4] *Brit. M. J.*, 1901, ii. 1345.

The nervous affections of the heart were the subject of the Morison Lectures by Dr. G. A. Gibson.[1] After a survey of the clinical aspects of the sensory affections of the heart, and an historical outline of the subject, he enters a protest against the separation of the angina into true and false, and considers that Balfour's arguments on this question are unanswerable. The commonly accepted views are embodied in Huchard's table[2]:—

| True. | Pseudo-Angina. |
|---|---|
| Most common between 40 and 50 years. | At every age, even six years. |
| Most common in men. Attacks brought on by exertion. | Most common in women. Attacks spontaneous. |
| Attacks rarely periodical or nocturnal. | Often periodical and nocturnal. |
| Not associated with other symptoms. | Associated with nervous symptoms. |
| Vaso-motor form rare. Agonizing pains and sensation of compression by a vice. | Vaso-motor form common. Pain less severe: sensation of distension. |
| Pain of short duration. Attitude: silence, immobility. | Pain lasts one or two hours. Agitation and activity. |
| Lesions: Sclerosis of coronary artery. | Neuralgia of nerves and cardioplexus. |
| Prognosis grave, often fatal. | Never fatal. |
| Arterial medication. | Anti-neuralgic medication. |

He embraces all the varieties of sensory defects of the heart under the one term angina, including sensations of vague uneasiness, numbness or deadness, feelings of cold and heat, sense of oppression or constriction amounting to excruciating agony, with sinking and a sense of impending death,—sensations distributed widely over the first six thoracic segments of the cord, or limited to pericardium and epigastrium, or radiating therefrom to the angle of scapula, shoulders, ulnar aspects of the arm and fore-arm to the extremities of fingers; also to the inguinal regions, into the head and neck, and including what is known as globus.

"It is earnestly to be hoped," he writes, "that the term pseudo-angina will, before long, be banished from modern medicine. It ought to be relegated to the limbo of archaic notions, as an entire anachronism in the twentieth century of our era." The division of things so widely different from each other into two great classes, the organic and inorganic, is admissible, but no absolute line can be drawn between even these two great classes.

"Amongst the organic type must be grouped instances of the affection dependent upon alterations about the origin of the aorta; changes in the walls and lumen of the coronary arteries; modification in the structure of the myocardium, of which fibroid, fatty, pigmentary, and gummatous lesions are the most common; the presence of pericarditis; the existence of aneurism; the growth of tumour.

[1] *Edin. M. J.*, 1902, N. S., xii. 24.
[2] Osler, *The Principles and Practice of Medicine*, 4th Ed., 1901, p. 764.

"The inorganic varieties fall into toxic and neurotic classes. The former, or toxic class, may be due to chemical or microbic influences. Amongst the former, tobacco certainly holds the foremost place; but alcohol, in this country, is certainly responsible for a considerable proportion of cases; tea, on the other hand, judging by my own observations, is an extremely rare cause of painful cardiac symptoms; certain of the endogenous chemical poisons are also potent, more particularly those which are connected with the symptoms commonly classified under the terms gout and diabetes. Amongst the microbic factors, influenza is certainly the most powerful, but malaria, diphtheria, and typhoid fever also furnish instances. The latter, or neurotic group, is a somewhat indefinite class; it practically resolves itself, however, into four varieties. There are cases in which reflex agencies are the cause of the symptoms; in which vasomotor instability is the dominant factor; in which neurasthenia forms the determining condition; and lastly, in which hysteria is responsible for the onset of the affection."[1]

R. SHINGLETON SMITH.

**Tubercular ulcers of the stomach** are rare, and their symptoms are often marked by those of widespread tubercular disease. Batsère in a series of observations[2] discussed their diagnosis. They are generally multiple and situated near the pylorus on the greater curvature. Instead of the irregular shape of tubercular ulcers of the bowels, these ulcers are round. They may cause severe lancinating pain upon taking food, which sometimes goes on to vomiting and hæmatemesis. The latter is of course strong evidence of the condition, if other causes can be excluded. The progress is steadily downhill in most cases; but occasionally the symptoms disappear, though this may be followed by severe hemorrhage after an interval.

Hæmatemesis in uræmia, appendicitis, acute intestinal stenosis, and from minute erosions should also be remembered. Hale White[3] thinks that many cases in chlorotic girls are due to erosions rather than to real ulcers, and remarks on the recurrence of these bleedings in some women until the menopause, and the fact that ulcers have occasionally been absent in such patients when the stomach has been opened by surgeons. However, the diagnosis from ulcer is most obscure clinically.

A very interesting research on the so-called **peripheral reflex secretion of the pancreas** by Bayliss and Starling[4] has disclosed the fact that the mucous membrane of the duodenum and jejunum secretes a substance, under the influence of the acid chyme from the stomach, which excites through the blood a copious secretion from the pancreas. There is nothing reflex

[1] Gibson, *loc. cit.*

[2] *Thèse de Toulouse*, 1901; Abstract in *Brit. M. J.*, 1902, i. Epitome, p. 37.

[3] *Lancet*, 1901, i. 1819.

[4] *Vide infra*. *Proc. Roy. Soc. Lond.*, Jan. 23.

about the process, for it takes place when all the nerves are removed. The "secretion" is split off from an inert prosecretion by the H.Cl., and is apparently a body of definite composition and of small molecular weight. A sympathy between different organs, such as the uterus and mammæ, has been often assumed; but this is the first instance where direct chemical proof has been obtained and the process explained. In the Lausanne laboratory[1] a somewhat similar research has shown how the stomach manages to secrete the quantity of acid or pepsin required for a given meal. It turns out that certain substances when ingested cause an increased secretion of the agent required for digestion. Thus the secretion of the H.Cl. can be greatly increased by small doses of meat extract or of ethyl alcohol, that of pepsine by minute doses of dextrin, though it is checked by large ones. The action takes place in some cases at least through the blood stream, for dextrin injected into the rectum will increase the output of pepsin in the stomach.

G. PARKER.

